# Rare cutaneous manifestations of statin-induced anti-3-hydroxy-3-methylglutaryl-coenzyme A reductase immune-mediated necrotizing myopathy

**DOI:** 10.1016/j.jdcr.2026.07.073

**Published:** 2026-08-29

**Authors:** Manon Blaise, Yann David, Villa Luisa, Anne-Flore Albertini, Edoardo Malfatti, Francisca Fajre, Fanny Rocher, Justine Baudron, Yoann Di Filippo, Michela Carlon

**Affiliations:** a Department of Dermatology, Hospital of Grasse, Grasse, France; b Department of Neurology, Hospital of Grasse, Grasse, France; c Department of Neurology, University Hospital of Nice, Nice, France; d Department of Pathology, Medipath, France; e Reference Center for Neuromuscular Disorders, AP-HP Henri-Mondor University Hospital, Créteil, France; f University Paris Est Créteil, Créteil, France; g Department of Pharmacology and Pharmacovigilance Center of Nice, University Hospital of Nice, Nice, France; h Department of Dermatology, Hospital of Cannes, Cannes, France; i Department of Gastroenterology, Hospital of Grasse, Grasse, France

**Keywords:** anti-HMCGR, cutaneous manifestations, immune-mediated necrotizing myopathy, statin-induced myopathy

Anti-3-hydroxy-3-methylglutaryl-coenzyme A reductase (HMGCR) immune-mediated necrotizing myopathy (IMNM) is a rare idiopathic inflammatory myopathy, most commonly presenting with severe muscle weakness and markedly elevated creatine phosphokinase (CK) levels.^1^ Anti-HMGCR–associated IMNM has been strongly linked to prior statin exposure and may persist even after statin discontinuation.^2^^,^^3^ Extramuscular manifestations of anti-HMGCR IMNM are uncommon. They are often overlooked because the predominant muscular involvement dominates the clinical picture. Unlike dermatomyositis, cutaneous involvement is considered rare and poorly characterized, and few cases have detailed histology.^4^ We report a rare presentation of statin-associated, anti-HMGCR–positive IMNM with atypical cutaneous involvement.

A 75-year-old man from the Philippines presented with progressive proximal muscle weakness, myalgia, dysphagia, and weight loss over several months. His medical history was notable for metabolic disorders including dyslipidemia, and monoclonal gammopathy of undetermined significance, and he had been treated with atorvastatin (20 mg/d) for approximately 4 years. Physical examination demonstrated symmetric weakness of the deltoids (Medical Research Council grade 3/5) and mild-to-moderate weakness of the finger extensors, iliopsoas, hamstrings, and quadriceps (Medical Research Council grade 4/5). The patient reported difficulty swallowing both solids and liquids, while respiratory function remained intact. Cutaneous examination revealed 2 right cervical erythematous plaques, slightly firm to palpation (Fig 1, *A*, *B*), present for approximately 6 months prior to the onset of muscle symptoms. The plaques were refractory to 1 month of daily treatment with potent topical corticosteroids (betamethasone dipropionate). No cutaneous findings suggestive of dermatomyositis were noted.

**Fig 1:**
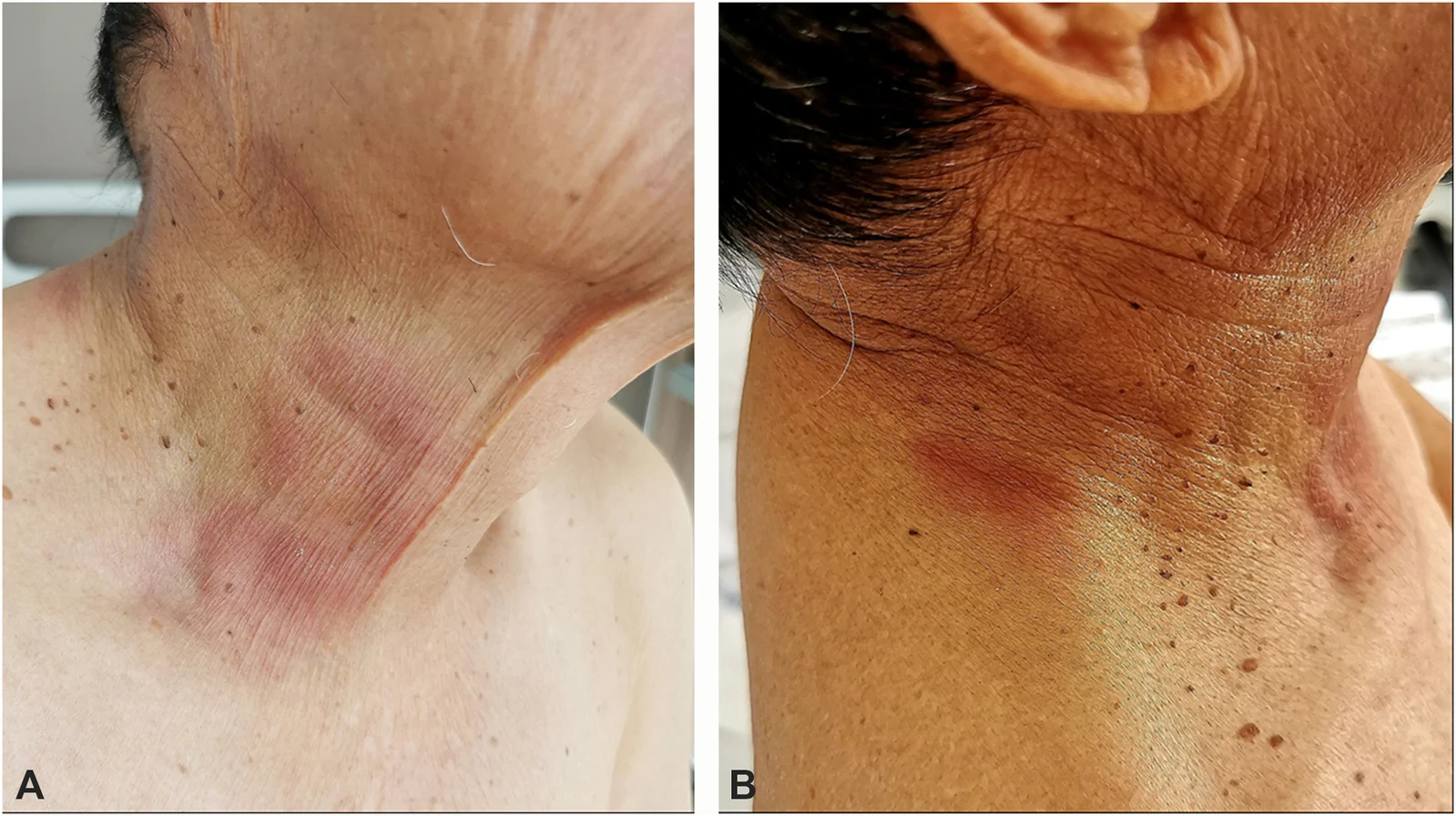
Initiation clinical presentation of the patient. **A,** Infiltrated lateral cervical plaque on palpation. **B,** Nuchal lesion developing secondarily to the lateral cervical lesion, with induration on palpation.

Laboratory evaluation revealed markedly elevated CK (5,677 IU/L; normal, <190 IU/L). Aspartate aminotransferase and alanine aminotransferase were also elevated (205 U/L and 299 U/L, respectively; normal, 10-35 U/L). C-reactive protein was 120 mg/L (normal, 0-5 mg/L). Antinuclear antibodies and anti-signal recognition particle antibodies were negative, whereas anti-HMGCR antibodies, detected by enzyme-linked immunosorbent assay (INOVA Diagnostics) were positive with a level of 193 U/mL (normal, <20 U/mL). Electromyography demonstrated myogenic patterns in several muscles of both upper and lower parts of the limbs (eg, the left extensor digitorum communis and the right brachioradialis) (Fig S1, *A, B*, available via Mendeley at https://doi.org/10.17632/dw8vxgp96h.1). Moreover, diffuse spontaneous activity (positive sharp waves and fibrillation potentials), consistent with ongoing denervation, was observed (Fig S1, *C, D*, available via Mendeley at https://doi.org/10.17632/dw8vxgp96h.1). Quadriceps muscle biopsy showed numerous necrotic and regenerating fibers with minimal inflammatory infiltrate, consistent with IMNM. Perifascicular atrophy was absent, and endomysial inflammation was minimal. Major histocompatibility complex class I expression was diffusely upregulated on affected fibers (Fig 2, *A-C*). Skin biopsy of the cervical plaque revealed a sparse pericapillary and interstitial dermal lymphohistiocytic infiltrate, without epidermal or dermoepidermal junction involvement and no evidence of granulomatous inflammation (Fig 3, *A-C*). Direct immunofluorescence was negative. The combination of clinical, laboratory, electromyographic, serological, and muscle biopsy findings fulfilled the European Neuromuscular Centre 2016 consensus criteria for definite IMNM,^5^ characterized by subacute proximal muscle weakness, marked hyperCKemia, anti-HMGCR antibody positivity, and muscle biopsy showing prominent myofiber necrosis and regeneration with minimal inflammatory infiltrate. These findings established the diagnosis of statin-associated, anti-HMGCR–positive IMNM with atypical cutaneous involvement. Oral corticosteroids (1 mg/kg) combined with intravenous immunoglobulins (2 g/kg over 5 days) were initiated as first-line therapy to achieve rapid disease control while completing the preimmunosuppressive workup. This combination led to significant biochemical improvement and marked resolution of the cutaneous lesions, but only modest gains in muscle function. Azathioprine was subsequently introduced as a maintenance immunosuppressive agent.

**Fig 2:**
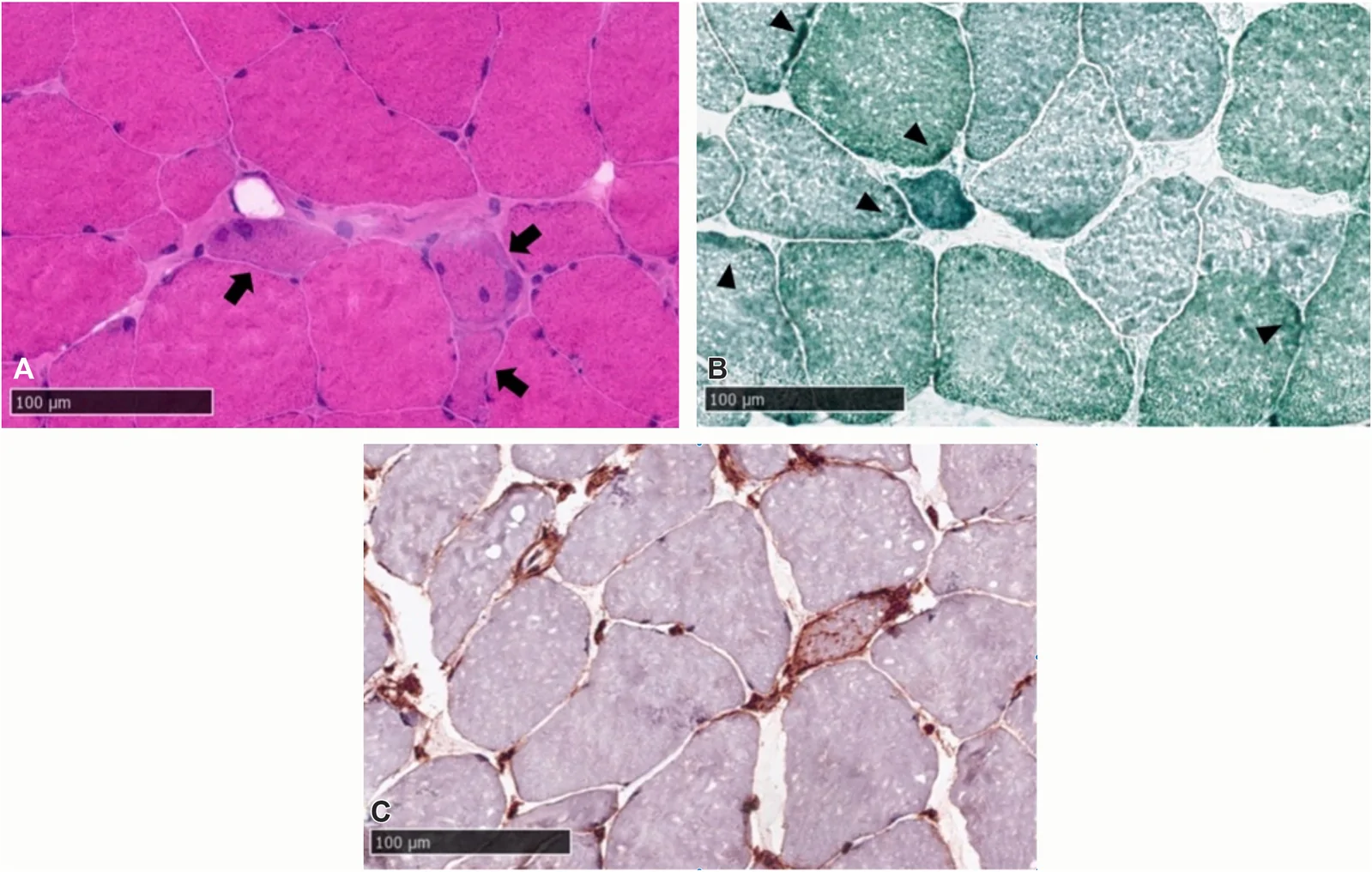
Muscle biopsy. **A,** Hematoxylin and eosin shows fiber size variability with regenerating fibers (*arrow*). **B,** Succinate dehydrogenase shows subsarcolemmal mitochondrial aggregates in some fibers (*arrowhead*). **C,** Human leukocyte antigen (HLA) including HLA-A, HLA-B, and HLA-C shows multifocal sarcolemmal overexpression of HLA class I.

**Fig 3:**
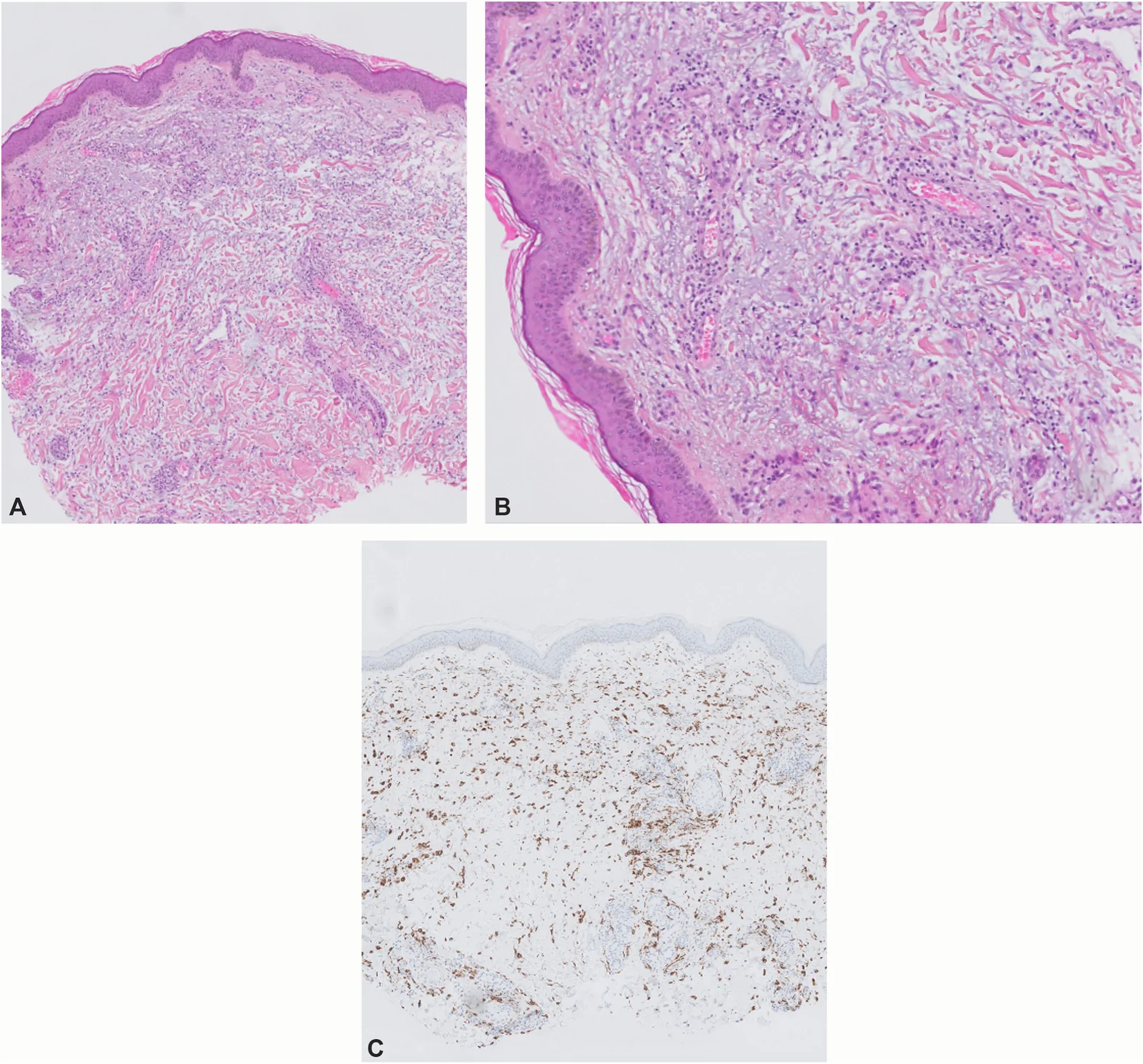
Histopathologic findings from the right lateral cervical skin lesion. **A** and **B,** Dense dermal inflammatory infiltrate, predominantly lymphocytic, with numerous histiocytes showing perivascular cuffing and interstitial distribution. The epidermis and dermoepidermal junction are spared. No collagen alteration of mucin deposition is observed. **C,** Immunohistochemical staining for CD68 demonstrates a histiocytic component within the subinterstitial dermal infiltrate. (**A** and **B,** Hematoxylin-eosin; original magnifications: **A,** á2; **B,** á10; **C,** á2).

Anti-HMGCR IMNM is a subtype of idiopathic inflammatory myopathy, classically characterized by severe proximal muscle weakness, markedly elevated CK levels, and predominant muscle fiber necrosis with limited inflammatory infiltrate on muscle biopsy. Extramuscular manifestations are generally considered uncommon, and cutaneous involvement has historically been reported in less than 10% of cases, including nonspecific rashes, Raynaud phenomenon, and occasional dermatomyositis-like eruptions.^6^^,^^7^

Scard et al^6^ reported a dermatomyositis-like cutaneous presentation in statin-induced anti-HMGCR IMNM, featuring erythematous and nodular lesions involving the back, upper portion of the arms, and legs. Skin biopsy revealed a nonspecific interface dermatitis with basal vacuolar change and scattered necrotic keratinocytes.^6^ Lim et al^8^ described statin-associated anti-HMGCR IMNM with erythematous to violaceous plaques predominantly affecting photo-exposed areas, including upper portion of the chest and upper extremities. The distribution and morphology of the lesions were considered clinically suggestive of dermatomyositis, although no pathognomonic cutaneous signs of classic dermatomyositis were definitively identified. Cutaneous biopsy revealed a nonspecific interface dermatitis with basal vacuolar change, scattered necrotic keratinocytes, and a sparse superficial perivascular lymphocytic infiltrate, lacking the characteristic histopathologic hallmarks of dermatomyositis.^8^ Furthermore, Parikh et al^9^ observed dermatomyositis-like violaceous papules and plaques on the hands and elbows. Cutaneous histology revealed a nonspecific interface dermatitis with basal vacuolar change and scattered necrotic keratinocytes, associated with a sparse superficial perivascular lymphocytic infiltrate without definitive histologic hallmarks of dermatomyositis.^9^ Kurashige et al^7^ retrospectively analyzed 88 patients with idiopathic inflammatory myopathies and found that 42% of patients with HMGCR-IMNM exhibited atypical skin lesions, predominantly on the neck and back, whereas patients with other myositis subtypes rarely showed skin involvement (*P* < .001). Lesions included nonspecific erythematous patches, nodular lesions, or ash-like scaly eruptions, and skin biopsies revealed Bcl-2−positive lymphocytic infiltrates similar to those observed in muscle biopsies, suggesting a shared immunopathologic mechanism. The mean age at diagnosis for HMGCR-IMNM patients was 48.4 ± 19.7 years. Among patients with skin involvement, symptom onset tended to occur at a younger age than in those without cutaneous manifestations. Approximately 37% of HMGCR-IMNM patients had prior exposure to statins, although the study did not provide detailed information regarding the specific type of statins or duration of exposure. The chronology of skin versus muscle involvement was not systematically reported; however, the overall disease duration in HMGCR-IMNM patients was longer (mean, 70.9 months) than in other IMNM subtypes, reflecting the often insidious course of this condition. Lesions included nonspecific erythematous patches, nodular lesions or ash-like scaly eruptions, and skin biopsies revealed Bcl-2−positive lymphocytic infiltrates similar to those observed in muscle biopsies, suggesting a shared immunopathologic mechanism.^7^ These findings highlight that cutaneous manifestations in HMGCR-IMNM are heterogeneous, can precede muscle involvement, and may not resemble classic dermatomyositis.

Dermatologists should be aware that IMNM may present with skin findings, which can be misinterpreted as unrelated dermatologic conditions. Cutaneous involvement can occur in statin-associated, anti-HMGCR−positive IMNM, even in the absence of classical dermatomyositis features. Awareness of the atypical presentations is important for prompt diagnosis and management.

## Conflicts of interest

None disclosed.
